# Predicting cardiovascular disease among diabetic patients in Ethiopia using machine learning models: evidence from Ethiopian public health Institute data (2024/2025)

**DOI:** 10.1186/s12889-025-24850-2

**Published:** 2025-11-24

**Authors:** Ashagrie Anteneh, Muluken Belachew, Sefefe Birhanu, Maru Meseret

**Affiliations:** Debre Markos University, Amhara, Ethiopia

**Keywords:** Cardiovascular disease, Machine learning, Diabetes, Gradient boosting model

## Abstract

**Introduction:**

Cardiovascular disease (CVD) is the leading cause of death among individuals with diabetes, accounting for nearly 50% of diabetes-related mortality. In Ethiopia, the burden of diabetes is increasing, yet there is a lack of predictive tools for identifying those at highest risk of developing CVD. In Ethiopia recent studies report a CVD prevalence of 37.26% among diabetic patients. This study employed machine earning to predict CVD among Ethiopia diabetic patients using Ethiopian public Health Institute (EPHI) datasets, with a focus on identifying the most influential risk factors for public health decision-making.

**Objective:**

The main objective of this study is to predict CVD among diabetic patients in Ethiopia using machine learning techniques.

**Method:**

The dataset comprised of 9030 instances with 22 features sourced from Ethiopian Public Health Institute. This prediction of cardiovascular disease (CVD) incorporated socio-demographic, behavioral, and clinical measurement data. Logistic regression, decision tree, Support Vector Machine, Random forest, Gradient boosting machine and artificial neural network were employed. Those models were trained on 80% of the data and tested on the remaining 20%. The analysis was conducted with python using 3.10.

**Results:**

According to the results analyzed, Gradient Boosting Model (GBM) demonstrated the highest overall performance, achieving an accuracy of 93%, followed closely by Logistic Regression (LR) with 90% accuracy. In terms of precision, GBM and LR performed comparably, while the LR achieved the highest recall at 88%. Regarding the F1 score, GBM attained 82%, indicating a strong balance between precision and recall. Additionally, the receiver operating characteristics (ROC) analysis showed that GBM had the largest area under the curve (AUC) of 0.96, reflecting superior descriptive ability 0.96.

**Conclusion:**

The gradient boosting machine (GBM) model demonstrated the highest performance compared to the other models, achieving an accuracy of 93%. The most significant factors influencing the GBM model were total cholesterol, hypertension, and fasting blood glucose levels. The gradient boosting model shows potential for future integration into clinical decision-support systems, pending external validation and early prediction of cardiovascular disease in individuals with diabetes.

## Introduction

### Background of the study

Diabetes is a chronic medical condition marked by elevated blood glucose levels, which occurs when the body cannot produce enough insulin [1]. According to the international diabetes federation, 463 million individuals (aged 20–79) were diagnosed with diabetes in 2019, and it is anticipated that this figure will rise to700 million by 2045 [2]. The persistent nature of this condition leads to complications that may result in the dysfunction and failure of vital organs, such as the kidneys, eyes, and heart [3]. Cardiovascular disease stands as the leading cause death and illness among individuals with diabetes [4]. As a result, those with diabetes face a significantly higher risk of developing cardiovascular disease compared to those without such condition [5]. Elevated blood glucose levels in diabetic individual can cause vascular damage, which may lead to severe cardiovascular complications that account for 80% of mortality in diabetes cases. Coronary artery disease (CAD), heart failure, and stroke are responsible for 50% of deaths among diabetes mellitus patients [6].

Cardiovascular disease (CVD) is the leading cause of mortality globally, accounting for over 17.9 million deaths annually, with approximately 80% of these deaths occurring in low- and middle-income countries [7]. Cardiovascular disease encompasses various conditions that impact the heart and blood vessels, including, strokes, which damage the blood vessels that supply blood to the brain. Coronary artery disease, which obstructs the arteries that deliver blood to the heart, and peripheral artery disease, which affects blood vessels supplying blood to the legs and feet [8]. Several risk factors significantly contribute to the incidence of CVD including, hypertension, smoking, alcohol consumption, physical inactivity, age, and high body mass index. Furthermore, diabetes plays a critical role in exacerbating the progression of atherosclerosis, hypertension, and dyslipidemia, all of which are pivotal in the development of cardiovascular diseases [9]. The metabolic disturbance associated with diabetes further elevates the risk of CVD among affected individuals [10]. Diabetes can manifest as either type one or type two. Type one diabetes usually occurs in children, and type two diabetes occurs in adults [11]. Patients with type two diabetes face a two-to fourfold increased risk of developing cardiovascular diseases compared to non-diabetics [12]. predicting disease based on early-stage symptoms presents significant challenges for physicians in the medical field [13].

In contemporary health care, large amounts of data are generated, which cannot be effectively analyzed or interpreted using conventional statistical methods [14]. However, the emergence of advanced techniques such as machine learning offers remarkable enhancement and opportunity to alleviate the burden on the physicians, thereby improving accuracy, prediction, and the overall quality of care [15]. Machine learning models are capable of accomplishing this difficult task and can be of tremendous assistance in the early diagnosis and prediction of cardiovascular disease. Medical Machine presents numerous opportunities such as the discovery of hidden patterns that can be utilized to generate diagnostic accuracy on any medical dataset [16]. We utilized six machine learning models: Logistic regression (LR), decision tree (DT), random forest (RF), support vector machine (SVM), gradient boosting machine (GBM), and artificial neural network (ANN). Logistic regression serves as a statistical model for binary classification. Decision trees split data based on features to form a tree-like structure, useful for interpretability. Random forest generates multiple decision trees and averages their outputs to improve accuracy. SVM create hyper planes for data classification and perform effectively with high-dimensional features. GBM constructs an ensemble of weak learners to iteratively minimize errors. ANN emulates structure of the human brains and is adept at learning intricate, non-linear relationships. These models were chosen for their respective advantages in predictive healthcare modeling [17].

Established clinical tools have been utilized to evaluate the risk of cardiovascular disease (CVD); however, their dependence on manual processes and limited accessibility across healthcare facilities presents considerable challenges [18]. Physicians frequently rely on their knowledge and experience for prognosis and risk assessment, which may introduce bias and errors, ultimately undermining the quality of patient care [19]. This highlights the necessity to develop machine learning models specifically aimed at predicting cardiovascular diseases in diabetic patients in Ethiopia. Although the country has been slow to widely adopt these advanced methodologies, there exists a significant demand for the application machine learning [20].

Despite the wealth of literature concerning cardiovascular diseases, much of it is based on traditional statistical models and tends to concentrate on general cardiovascular conditions, failing to adequately considering diabetes as comorbidity. Consequently, this study seeks to address these gaps by developing machine learning techniques to analyze risk factors and identify the most prevalent and significant contributors [21]. This research will provide numerous benefits, particularly in enhancing prediction accuracy. Patients often endure their illnesses for prolonged periods without displaying symptoms, resulting in a scenario where, by the time they recognize the symptoms and effects, it is frequently too late [22]. Nevertheless, the integration of machine learning models in healthcare can assist in averting such situations by promoting early detection and precise prediction. Identifying at risk individuals enables healthcare professionals to undertake proactive measures and initiate timely treatments.

## Literature review

### Global evidence on cardiovascular diseases among diabetic patients

The growing availability of electronic health records and clinical data has significantly enhanced the potential for disease prediction through computational methods [14]. Despite this, predicting disease such as cardiovascular disease (CVD) in patients with diabetes remain a challenge, particularly at early-stages when clinical symptom may be absent or vague [13]. Machine learning(ML) has emerged as robust tool to bridge this gap by enabling the development of predictive models that can uncover hidden pattern and intricate association among clinical variables Habehh & Gohel, 2021 [15].

Several international studies have evaluated the effectiveness of different ML algorithms in predicting CVD among individuals with patients. For instance, Sang et al., 2024 [16] applied Logistic regression, support vector machine, extreme gradient boosting and random forest to an online dataset. They reported that the random forest algorithms achieved the best performance, with an AUC-ROC of 0.830. However, their model did not incorporate key life style-related risk factors such as alcohol consumption, smoking, physical activities.

In other study, Ranjani & Tamilselvi, 2024 [23] integrated biomarkers and electrolyte data into their models, finding that the XGBoost algorithm achieved the highest prediction accuracy of 0.82, outperforming random forest and logistic regression. Their results also highlighted the importance of ensemble learning techniques in capturing complex, multifactorial risks.

Nrusimhadri et al., 2024 [24] compared multiple algorithms, including decision trees, AdaBoost, support vector machines, artificial neural networks (ANN), and a customized ANN. The customized ANN yielded the highest accuracy at 94%, demonstrating that model architecture optimization can significantly influence predictive outcomes. were compared to predict cardiovascular diseases with the accuracy of as: Decision trees was 73%, AdaBoost was 78%, SVM was 72% accurate, ANN was 81% and CANN was 94% accuracy, revealed that the convolutional neural network (CNN) achieved the highest accuracy outperforming the other the algorithms.

Similarly, Mayya & Solieman 2022 [25] applied SMOTE (Synthetic Minority Oversampling Technique) to address class imbalance, improving performance in model such as random forest and XGBoost, which achieved accuracies above 90%.

Collectively, these studies underscore the growing interest in using ML to predict CVD risk in diabetic patients. Yet, many still overlook key behavioral and dietary factors such as alcohol, fruit, and vegetable consumption that may enhance model performance. Incorporating such variables could improve prediction accuracy and clinical relevance.

### Cardiovascular diseases among diabetic patients in Ethiopia

Ethiopia is currently experiencing an epidemiological transition, marked by rising rate of non-communicable (NCDs), including diabetes and cardiovascular diseases. Rapid urbanization and sedentary lifestyle and dietary shift are key contributors to this trend [26]. However, most studies conducted in Ethiopia have relied on classical statistical methods, with few incorporating machine learning approach to predict CVD among diabetic population.

Past studies identified hypertension, dyslipidemia, obesity, physical inactivity, and smoking as major risk factors for CVD among Ethiopian diabetic patients. For example, Tamiru & Alemseged, 2011 [27] reported prevalence rate of 46.5% for hypertension, 23.4% for dyslipidemia, 63.5% for obesity, 55.1% for physical inactivity, and 5.5% for smoking. Similarly, Abdosh and Weldegebreal, 2019 [9] identified high rate of dyslipidemia(90.6%), hypertension(62.7%), family history of CVD(8.2%),and physical inactivity(76%) in diabetic populations.

Further evidence from Debele, 2019 [28] linked chronic kidney disease and elevated triglyceride levels to increased CVD risk. According to G. R. Debele (2021), diabetic patients with systolic hypertension had more than four times the risk of developing CVD compared to those without it. Moreover, individuals with triglyceride levels above 200 mg/dL were found to be nearly five times more likely to develop CVD [29].

Despite the availability of such clinical insight, predictive tools incorporating these variables using modern machine learning techniques remain limited in Ethiopia. recent systemic reviews, such as that by Ayalew et al. 2023 [6], estimated the pooled prevalence of CVD among diabetic patients in Ethiopia at 37.26%. However, these reviews did not explore predictive modeling approaches or evaluate the relative importance of different risk factors.

### Research gaps and rationale

While existing global studies have successfully applied machine learning to predict cardiovascular disease (CVD) in diabetic populations, many have omitted key modifiable risk factors relevant to low-resource settings. In Ethiopia, although descriptive studies have identified important predictors of cardiovascular disease, there remains a lack of machine learning-based risk prediction models that can be integrated into clinical or public health practice. This study addresses this gap by developing and evaluating multiple machine learning models using nationally representative data from Ethiopia. It uniquely incorporates a wide range of variables-including behavioral, clinical, and socio-demographic factor to predict CVD in diabetic patient. The objective is not only to compare model performance but also to identify the most influential predictors of CVD, thereby supporting risk stratification and informing targeted interventions in the Ethiopian context.

### Research questions

Finally, the study answered the following question.

RQ1. Which model was the best effective to predict cardiovascular diseases among diabetic patents in Ethiopia?

RQ2. Which features were the most important risk factors for cardiovascular diseases among diabetes in Ethiopia?

## Objectives

### General objective

To predict cardiovascular diseases among diabetic patients using machine learning models in Ethiopia.

### Specific objectives

- To evaluate the models on the test set using the metrics such as Accuracy, Precision, Recall, F1 Score, and ROC curve.
- To compare the performances of these models to identify the most efficient algorithm.
- To identify significant features contributing to develop cardiovascular diseases among diabetic patients.

## Methods

In this study, our aim was to develop machine learning models that can predict cardiovascular disease among individuals with diabetes. The research followed a systematic approach that encompassed data collection, data preprocessing, model selection, and evaluation. The methodology employed is detailed in the following steps.

### Dataset

The dataset used in this study consist 9030 medical records of diabetic patients, obtained from Ethiopian Public Health Institute (EPHI) after securing ethical clearance. It included socio-demographic variables (age, sex, marital status, ethnicity, education attainment, and residential location), behavioral factors (smoking, tobacco use, alcohol use, khat consumption, physical activity, and dietary habits), and Clinical parameters (systolic and diastolic blood pressure, total cholesterol level, fasting glucose and triglyceride and BMI). The target variable was the presence or absence of cardiovascular disease (CVD) diagnosis, as documented in the patients’ medical records, where patients with coronary artery disease, heart failure or stroke were coded as having any of these conditions labeled as CVD = 1, while those without such diagnoses were labeled as CVD = 0.

### Data pre-processing

The technique of preprocessing utilized to prepare, cleanse and organize raw data is crucial for the development and training of machine learning models, which ultimately aim to enhance the model’s accuracy [30]. This process involved various methods, such as feature selection, data encoding, feature scaling,

#### Feature selection

Feature selection was utilized to determine the most pertinent features. To identify relevant features, Pearson’s’ correlation coefficient (r) was calculated between each independent variable and the dependent variable. Features with |r| >=0.01 were included in the analysis, as this threshold signifies at least a weak-to-moderate association.

**Fig. 1:**
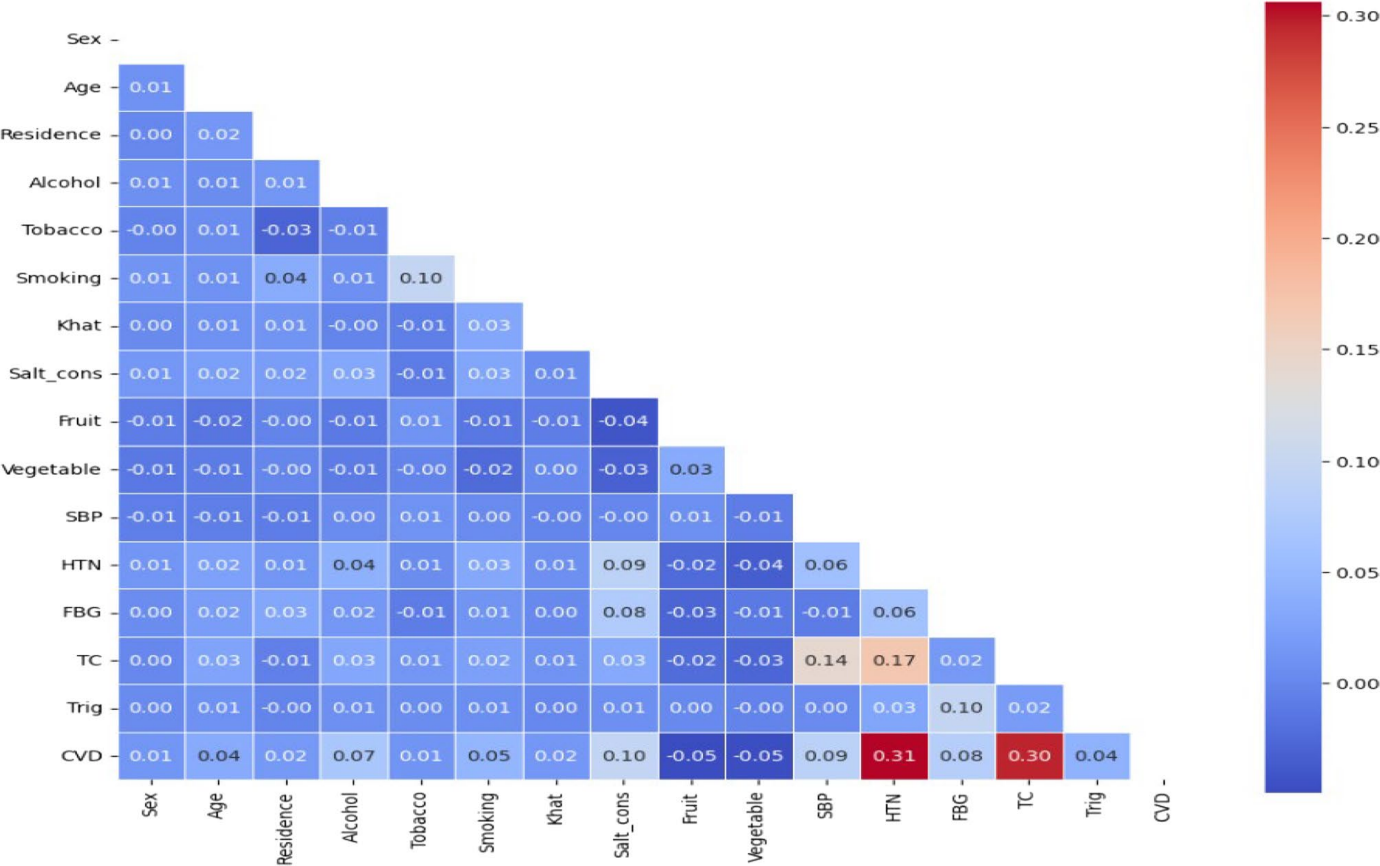
Heat map plot showing the correlations among selected features of cardiovascular disease among diabetes

Figure 1 demonstrated that cardiovascular disease (CVD) revealed the strongest positive correlation with hypertension (HTN), with a correlation of coefficient 0.31. Furthermore, CVD exhibited a high correlation with total cholesterol (TC), with a correlation of 0.32. Most clinical factors are significantly correlated with CVD. In contrast, most categorical features showed a weak correlation with CVD compared to numeric features. We employed a filter method to select features using a correlation matrix, where a cutoff point of |r| >=0.01 was deemed relevant for the study. To focus on features that are most predictive of the target, it was reasonable to remove features based on their correlation with the target Variable (CVD).

#### Data encoding

The dataset included categorical variables and numerical variables. Most algorithms perform more effectively when the data is represented in numerical form. Consequently, to transform categorical variables into a format that can be processed by machines, we employed encoding technique. Binary categorical variables (like sex: male/female) were transformed using liable encoding (male = 1, female = 0).Nominal categorical variables with more than two categories (like ethnicity, marital status) were processed using one-hot encoding to avoid introducing ordinal relationship.

#### Feature scaling

To normalize the range of instances within dataset, it is essential to implement feature scaling techniques. If the range differences among instance are significant, models may erroneously interpret large values as being more important, potentially resulting in inaccurate outcomes. To mitigate this problem, StandardScaler technique has been employed. Continuous variables, including age, body mass index (BMI), and blood pressure, were standardized to ensure comparable scales across feature. Standardization was performed using StandardScaler form scikit-learn which transform each feature as follows:

$$\mathrm z\;=\;\frac{\mathrm x\;-\;\mathrm\mu}{\mathrm\sigma}$$

​Where: 𝑥= original value of the feature, µ = mean of the feature, and 𝜎 = standard deviation of the feature.

Handling of missing values: The dataset was examined for missing entries across all variables. Numerical features with messing values were imputed using the mean of the respective feature, while categorical variables were imputed using the mode (most frequent category). Records containing more than 20% missing data were excluded from the analysis to minimize potential bias [21].

### Model development

We chose to employ various models, including Logistic regression, decision tree, random forest, support vector machine, gradient boosting machine and artificial neural network models. Logistic regression is predictive is the predictive analysis to conduct on discrete (binary) values based on a specified set of independent variables. LR describes the data and clarifies the relationship between one (binary) dependent variable and independent variables. Decision tree is a decision supporting tool that represented by a tree-like a graph or model of decision that represent the possible outcomes. Among these, Gradient Boosting and Random Forest are especially effective and commonly utilized for handling dataset that encompass both categorical and continuous variables. For datasets characterized high dimensionality, Support Vector Machine is a suitable option, classify data by separating the classes with a boundary, i.e. a line or multi-dimensional hyper plane [31]. Gradient Boosted Model (GBM) is also an ensemble prediction model based on decision trees. In contrast to Random Forest, this model successively builds decision trees using gradient descent in order to minimize a loss function. A final prediction is made using a weighted majority vote of all of the decision trees. We consider an implementation of gradient boosting, which is optimized for speed and performance [32].

Given the class imbalance present in the dataset, where only 18.5% of instances were positive for CVD, we applied the Synthetic Minority Oversampling Technique (SMOTE). This technique generates synthetic data points for the minority class to facilitate balanced learning. A threshold of 0.5 was upheld for classification following resampling. This strategy enhances model performance and mitigates bias toward the majority class.

### Training and testing models

Preprocessing techniques and label encoding were applied to the dataset. Subsequently, before training, the dataset was partitioned into training and testing sets in an 80:20 ratio. The training set consisted of 7224 rows and 22 columns, while the testing set included 1806 rows and 22 columns. To ensure the creation of robust predictive models with high accuracy, it is essential to select suitable hyper parameters. During the hyper parameter tuning process, each model was optimized using a Grid Search method to systematically evaluate different parameter combinations. For each fold, the training data was split into smaller training and validation subsets, ensuring that every data point contributed to both training and testing. The parameter ranges were chosen based on prior studies, domain knowledge, and initial exploratory trials. This approach helped mitigate over fitting, improve model generalization, and ensure that the selected hyper parameters maximized predictive performance on unseen data [33].

For logistic regression, we configured the solver to bilinear, with a maximum of 5000 iterations and a random state of 42. For Decision tree, the maximum depth of was established at 10, with a random state of 42. In the case of random forest, we utilized 10 estimators, with a maximum depth of 10, and a random state of 42. For support vector machine, the kernel was specified as linear, with a random state of 42. For Gradient boosting model, we set the number of estimators to 10, max depth to 5, and learning rate to 0.5. And For the artificial neural network, we utilized keras configuring various hyper parameter such as 50 epoch and the rectified leaner unit (ReLu) activation function for the hidden layers to introduce non-linearity into the network. Additionally, the Adam gradient decent optimization algorithm was employed as a solver, with the random state set to its default value of 42. (Tables 1 and 2) summarizes the tuned hyper parameters and their final selected values for the models used in this study.

**Table 1:** Tuned hyper parameter for each machine learning models

Model	Hyper parameter	Search Range	Selected Value
Logistic Regression (LR)	Solver	[‘lbfgs’, ‘saga’]	Lbfgs
	max_iter	[1000, 3000, 5000]	5000
	C	[0.01, 0.1, 1, 10]	1
Decision Tree (DT)	max_depth	[5, 10, 15, None]	10
	min_samples_split	[2, 5, 10]	2
Random Forest (RF)	n_estimators	[50, 100, 200]	100
	max_depth	[5, 10, 15, None]	10
	min_samples_split	[2, 5, 10]	2
Support Vector Machine (SVM)	Kernel	[‘linear’, ‘rbf’]	Linear
	C	[0.1, 1, 10]	1
Gradient Boosting Machine (GBM)	n_estimators	[50, 100, 200]	100
	max_depth	[3, 5, 7]	5
	learning_rate	[0.01, 0.1, 0.5]	0.5
Artificial Neural Network (ANN)	Epochs	[50, 100, 150]	50
	hidden_layer_sizes	[(64,), (128,), (64, 64)]	(64,)
	Activation	[‘relu’, ‘tanh’]	Relu
	Optimizer	[‘adam’, ‘sgd’]	Adam

**Table 2:** Summary statistics of numeric variables

	Age	Fri_cons	Veg_ cons	BMI	SBP	DBP	FBG	TC	Trig	HTN
count	9030	9030	9030	9030	9030	9030	9030	9030	9030	9030
Mean	47.7	2.8	2.9	25.1	134	85	125.8	193	150	0.2
Std	16.4	1.4	1.4	5	19	12	49.5	46	57.7	0.4
Min	15	1	1	10	110	70	72	89	50	0.0
25%	34	2	2	22	120	76	92	159	101	0.0
50%	49	3	3	25	130	83	112	182	150	0.0
75%	63	4	4	29	139	89	134	209	201	0.0
Max	70	5	5	40	189	119	170	329	250	1.0

### Model evaluation

The built models were evaluated on the test set with evaluation metrics such as accuracy, precision, recall, F1 score and AUC-ROC to identify the model that exhibits optimal performance in predicting cardiovascular disease (CVD). The dataset comprised 7360 instances of CVD (Yes = 1) and 1670 (No = 0) instances of Non-CVD resulting in an imbalanced class distribution. In this context, relying solely on accuracy may not provide a valid assessment, as it fail to differentiate between the correct classification of the various classes, potentially lead to incorrect conclusion [16]. Therefore, additional performance metrics, including, Recall, precision, F1 and ROC curve were utilized.

## Exploratory data analysis

Explanatory data analysis (EDA) is essential for comprehending the characteristic of data. Out of 9030 patients, 1670 (18.5%) were identified as having CVD (Yes = 1), while7360 (81.5%) were not (No = 0). Among the patients 4637(51.4%) were male, and 4393 (48.6%) were female. The mean age of the entire cohort was 47.7 years *(*SD *±* 16.5). Specifically, the mean age for individual identified with CVD was 50.9 years, in contrast to a mean age of 47 years for those without the condition. 45.2% females and 55.8% male were found to have CVD. The data indicates that majority cardiac patients’ were aged between 42 and 65 years. Additionally, the majority triglycerides levels range from 105 to 210 mg/dl, while fasting blood glucose (FBG) levels observed between 120 and 140 mg/dl. The Systolic blood pressure (SBP) measurements fall within the range of 105 and 145 mmHg, and diastolic blood pressure (DBP) values are between 77 and 92 mmHg. Furthermore, the most total cholesterol levels recorded between 225 and 275 mg/dl.

## Results

**Fig. 2:**
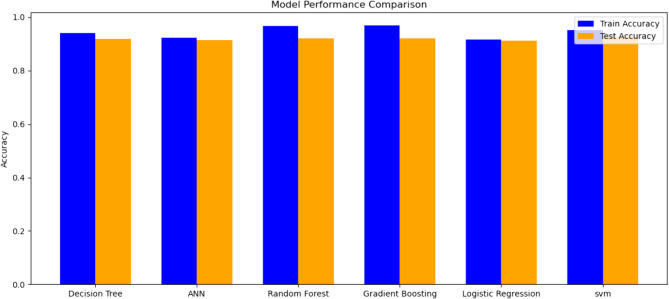
Training and testing accuracy of different models, illustrating their generalization performance

Across all models, hypertension, total cholesterol, and fasting blood glucose consistently emerged as the top predictors of CVD among diabetic patients. These factors were most significantly linked to an increased risk, as validated by the feature importance analysis of the gradient boosting model. The results for each model are outlined in Table 3, which display the values for each performance metric. According to the analyzed results, the Gradient Boosting Model achieved an accuracy of 93% with a precision of 80%, recall of 82%, F1-score of 83%, and an AUC of 0.96, outperforming other models (Tables 4 and 5). Among the models, Support Vector Machine (SVM) had the lowest performance with 90% accuracy and lower recall (67%) compared to GBM and RF Fig. 2.

**Table 3:** Summary statistics of categorical variables

	Sex	Ethnic	Residence	Smoking	Khat	Alchol	Salt	Physical
Count	9030	9030	9030	9030	9030	9030	9030	9030
Unique	2	11	2	2	2	2	2	3
Top	Male	Oromo	Rural	No	No	Yes	No	High
Freq	4637	2762	5465	7197	7119	5853	8233	4403

**Table 4:** Comparison of model performance before and after SMOTE using Accuracy, Precision, Recall, and AUC-ROC

Models	Accuracy (%)		Precision (%)		Recall (%)		F1-score (%)		AUC-ROC (%)	
	Before SMOTE	After SMOT	Before SMOTE	After SMOT	Before SMOTE	After SMOT	Before SMOTE	After SMOT	Before SMOTE	After SMOT
GBM	94	93	89	80	76	82	82	83	0.96	0.96
RF	94	92	92	78	75	82	82	78	0.96	0.95
LR	93	90	82	68	79	88	80	77	0.95	0.95
ANN	92	90	86	73	71	75	78	74	0.94	0.91
SVM	85	90	65	67	54	87	53	76	0.84	0.94
DT	92	90	77	72	78	79	78	75	0.87	0.86

**Table 5:** Training and testing losses of each model, indicating learning effectiveness and potential over fitting

Models	Train loss	Test loss		
LR	0.178651		0.174130	
DT	0.243851			0.536172
RF	0.148038			0.150200
GBM	0.090059			0.129120
SVM	0.3473916			0.364023
ANN	0.2348267			0.326768

## Discussion

This study developed and evaluated a machine learning models to predict cardiovascular disease (CVD) among diabetic patients in Ethiopian, using nationally representative data from Ethiopian Public Health Institute (EPHI). The analysis not only compares Machine learning models but also identifies risk factors relevant for cardiovascular disease.

Among the models evaluated, the Gradient Boosting Model (GBM) achieved the best overall performance with an accuracy of 93%, and AUC of 0.96. Logistic Regression (LR) and Random Forest (RF) also showed strong performance, with AUC values of 0.95 each. The Gradient Boosting Machine (GBM) showed relatively better performance in handling non- linear patterns in our datasets, which may explain its higher accuracy compared to the other models. Its iterative boosting framework, which reduces errors by integrating weak learners, enabled it to capture intricate, non- linear relationships present in the data.

**Fig. 3:**
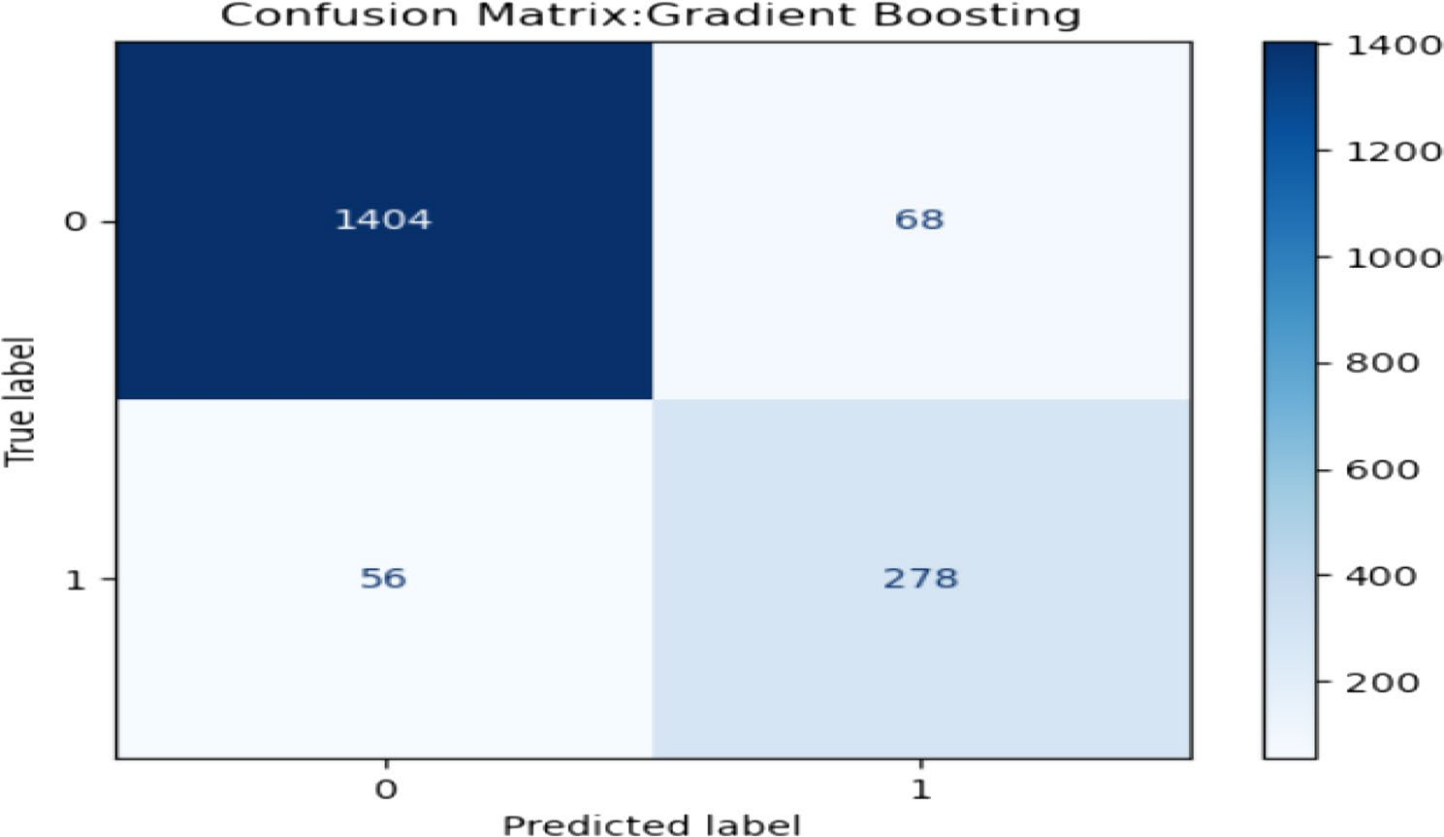
The confusion matrix of gradient boosting model predictions on the test dataset, illustrating True Positives (TP), False Positives (FP), True Negatives (TN), and False Negatives (FN)

The confusion matrix for the gradient boosting model, illustrated in Fig. 3, details the models’ prediction on the test dataset. Out of 1810 test samples, the model accurately identified 278 patients as having CVD and correctly classified 1404 patients as not having CVD. However, it mistakenly predicted 56 patients as having CVD and misclassified 68 patients as not having the disease.

**Fig. 4:**
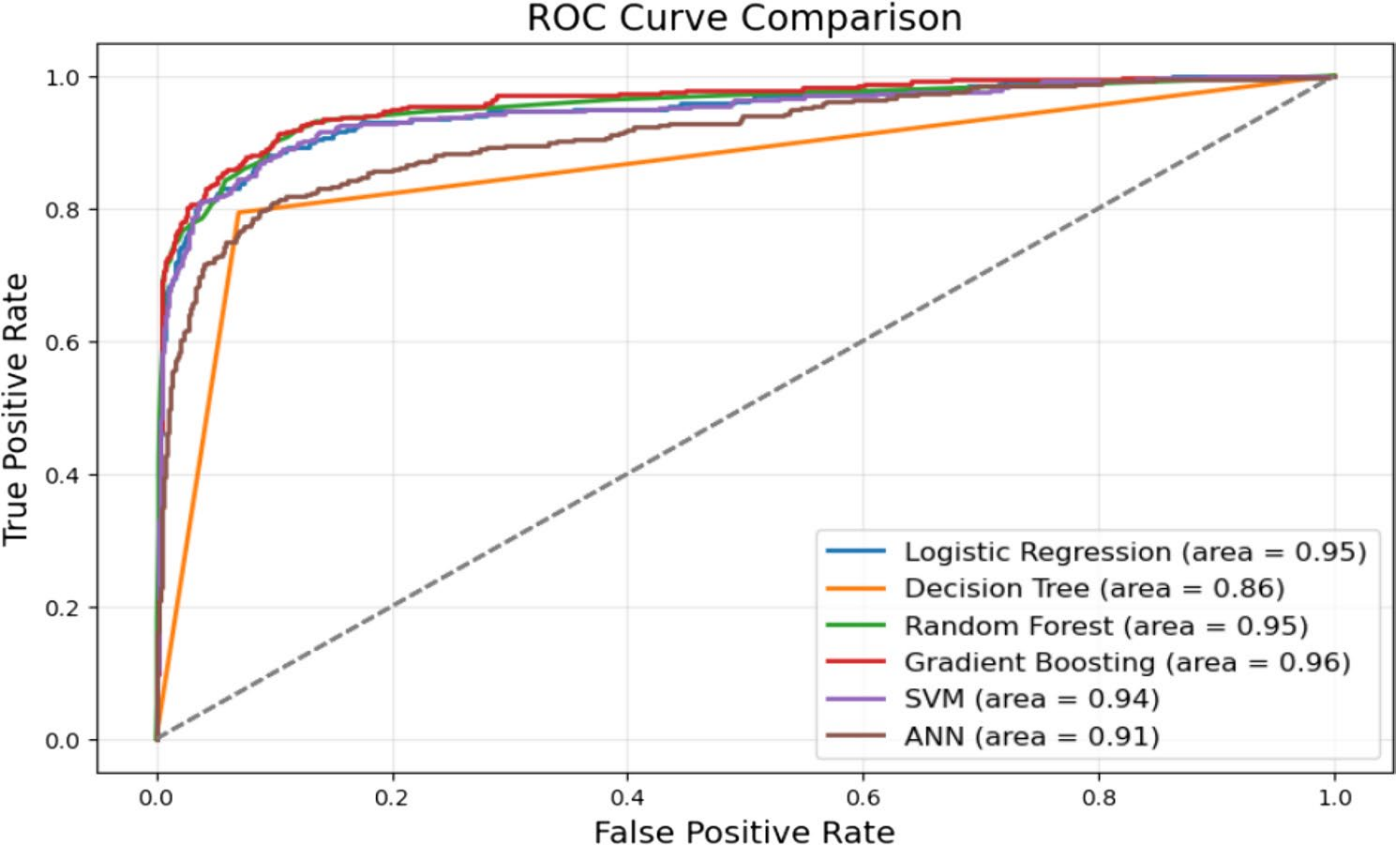
ROC curves for different machine learning models, comparing their ability to distinguish between patients with and without cardiovascular disease

In situations of data imbalance, accuracy alone may not be sufficient to evaluate model performance [25]. Therefore, additional metrics such as precision, recall, F1 score, and ROC curve analysis were employed. As observed, the GBM classifier excelled across these metrics, confirming it as the most effective model in this analysis. Figure 4 presents the ROC curves for all models, illustrating the trade-off between True Positive Rate (Sensitivity) and False Positive Rate. A model with a curve positioned in the upper-left corner indicates strong discrimination between classes.

**Fig. 5:**
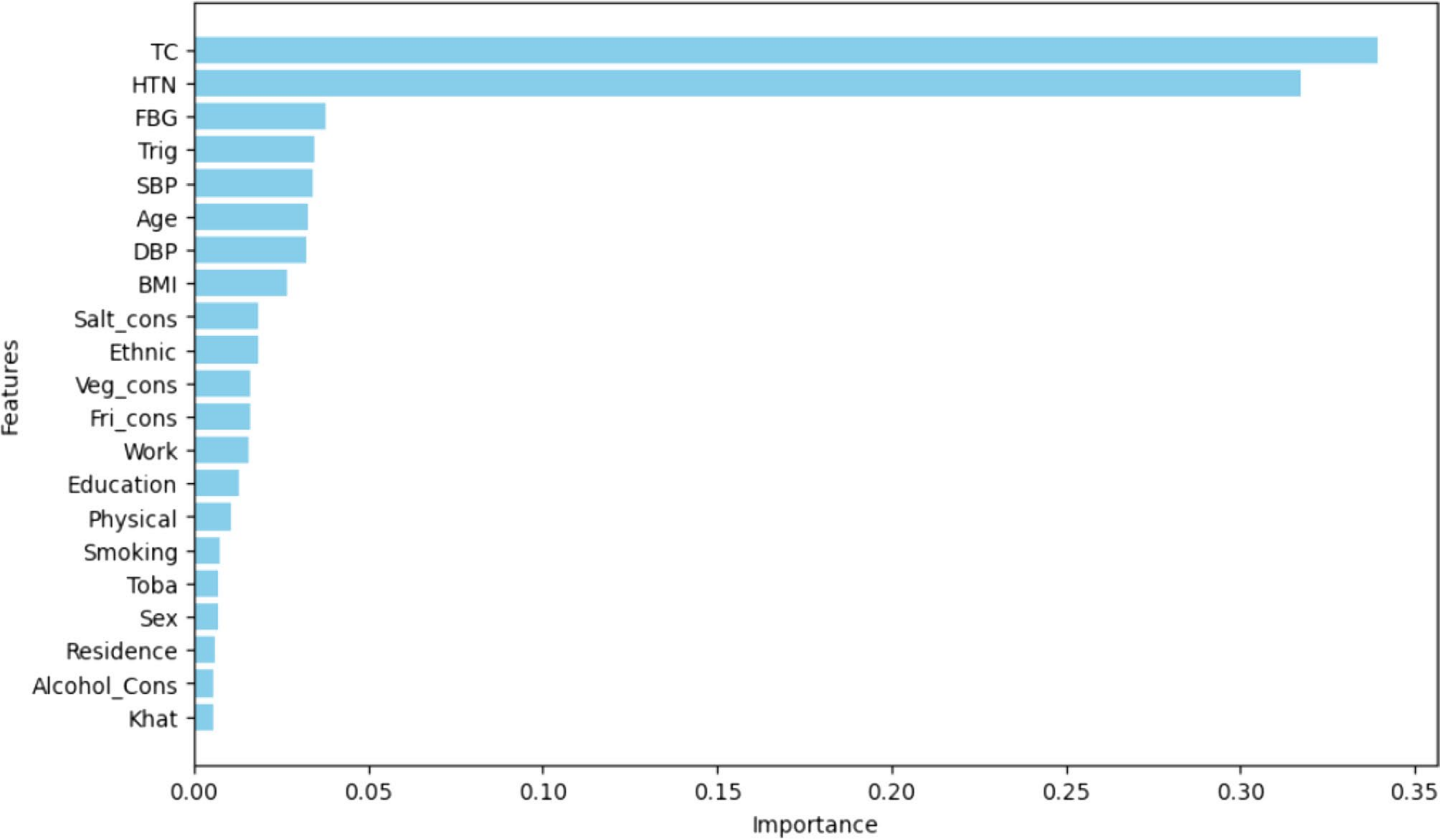
Feature importance ranking of gradient boosting machine, highlighting variables with the strongest influence on predicting cardiovascular disease

Feature importance analysis using GBM revealed that total cholesterol, fasting blood glucose, and hypertension were the most influential predictors for CVD (Fig. 5). A total of 21 features were analyzed, and the ranking of feature weights demonstrated that these variables held the greatest influence on model predictions. The lengths of the line segments in Fig. 5 indicate the relative impact of each feature.

The logistic regression model indicated a positive correlation between total cholesterol and systolic blood pressure with increased CVD risk, while regular fruit consumption was associated with a reduced risk. Specifically, the odds ratio for total cholesterol suggested that a one-unit increase was linked to a 1.5 times higher risk of CVD, after controlling for other variables.

In addition to cholesterol and blood pressure, diabetic patients with fasting blood glucose levels exceeding 125 mg/dl and triglyceride levels above 200 mg/dl were found to be at higher risk for developing CVD. Older age and higher body mass index were also significant contributors. Male patients were identified as having a higher risk of CVD, likely due to biological and behavioral factors such as higher rates of smoking, khat chewing, and alcohol consumption compared to females.

These findings are consistent with global studies, highlighting the widespread relevance of fasting blood glucose, triglycerides, cholesterol, and other risk factors in predicting CVD [28], Recognizing these indicators is crucial for early detection and intervention in both national and international public health strategies.

The performance of our models aligns with recent studies conducted in India [24]. Our AUC values (0.96 for GBM, 0.95 for LR and RF) were higher than those reported in Korean cohorts (0.81–0.84) [16]. and Polish datasets (0.62–0.72) [34]. The difference may be attributed to the larger sample size, inclusion of more behavioral variables, and variations in data preprocessing and feature selection methods. Larger datasets often provide more diverse and representative samples, enhancing the generalizability and robustness of the model, which leading to better performance.

This research represents one of the pioneering studies in Ethiopia that employs nationally representative data alongside advanced machine learning techniques to predict cardiovascular disease in individuals with diabetes. Incorporating both behavioral and clinical variables provides a thorough, data-driven approach to understanding CVD risk.

From the policy perspective identifying high-risk diabetic patients through machine learning models can facilitate early screening, targeted interventions, and optimized resource allocation. Integrating machine learning-based risk stratification tools into routine diabetes management could improve evidence-based decision-making and enhance patient outcomes at a population level.

## Conclusion and recommendations

Among the evaluated models, the GBM model achieved the best performance (accuracy 93%, AUC 0.96). Total cholesterol, hypertension and fasting blood glucose levels were identified as the strongest predictors of CVD among diabetic patients in Ethiopia. While these findings highlight the potential of machine learning models to support risk prediction, their integration into clinical practice will require further evidence.

Despite the robust performance of the model, certain limitations should be noted. Firstly, the study relied on secondary data, which lead to missing information and the exclusion of some important variables. Secondly, the cross-sectional design provided only a snapshot of the situation at a specific time point, limiting the ability to examine long-term effect of risk factors. Thirdly, the absence of external validation using independent datasets restricts the generalizability of the models as they were evaluated solely through internal train-test splits.

Future research should incorporate multi-center or longitudinal datasets for external validation to confirm clinical relevance. In addition, the use of model interpretability techniques such as SHAP or LIME is recommended to enhance transparency and facilitate clinical applicability.

## Data Availability

No datasets were generated or analysed during the current study.
